# THE RECIPROCAL RELATIONSHIPS AMONG SENSE OF PURPOSE, LONELINESS, AND COGNITIVE DECLINE

**DOI:** 10.1093/geroni/igae098.0554

**Published:** 2024-12-31

**Authors:** Gabrielle Pfund, Anthony Ong, Daniel Mroczek, Bryan James, Kathryn Jackson, Eileen Graham

**Affiliations:** Auburn University, Auburn, Alabama, United States; Cornell University, Ithaca, New York, United States; Northwestern University, Evanston, Illinois, United States; Rush University Medical Center, Chicago, Illinois, United States; Northwestern University, Evanston, Illinois, United States; Northwestern University, Evanston, Illinois, United States

## Abstract

Dementia is the leading cause of disability in older adults worldwide. As of yet, there are no easily accessible cures. Thus, a wide range of research has focused on modifiable intra- and interpersonal factors that may contribute to lower risk for dementia and generally slow down cognitive decline. A promising protective intrapersonal factor from the well-being literature is sense of purpose, as higher levels of sense of purpose are tied to a swath of healthy cognitive aging outcomes. Meanwhile, greater experiences of loneliness are typically a risk factor for poorer cognitive aging. The current study calls upon data from the ongoing Memory and Aging Project from the Rush Alzheimer’s Disease Center (N = 1,736; waves: up to 18; age: M = 81.07, SD = 8.04, 65-101). Every year, participants complete an extensive cognitive battery and report on their sense of purpose and loneliness. Given the likely reciprocal relationship between these three constructs, the current project uses random intercept cross-lagged panel models to better establish the between- versus within-person and concurrent versus longitudinal processes behind these relationships. Findings highlight the bidirectional relationship of these variables across time metrics and levels. For example, lower levels than one’s average of sense of purpose predicts decreases in cognitive functioning a year later, and decreases in cognitive functioning also precede decreases in sense of purpose. Future research would benefit from exploring combined efforts to bolster sense of purpose and reduce loneliness, while continuing to mind the potential influence of cognitive decline on these constructs.

